# StrucNet: a global network for automated vegetation structure monitoring

**DOI:** 10.1002/rse2.333

**Published:** 2023-04-14

**Authors:** Kim Calders, Benjamin Brede, Glenn Newnham, Darius Culvenor, John Armston, Harm Bartholomeus, Anne Griebel, Jodie Hayward, Samuli Junttila, Alvaro Lau, Shaun Levick, Rosalinda Morrone, Niall Origo, Marion Pfeifer, Jan Verbesselt, Martin Herold

**Affiliations:** ^1^ CAVElab – Computational & Applied Vegetation Ecology, Department of Environment Ghent University Coupure links 653 Ghent 9000 Belgium; ^2^ School of Forest Sciences, University of Eastern Finland Joensuu 80101 Finland; ^3^ Helmholtz Center Potsdam GFZ German Research Centre for Geosciences Section 1.4 Remote Sensing and Geoinformatics Telegrafenberg Potsdam 14473 Germany; ^4^ CSIRO Research Way Clayton Victoria 3168 Australia; ^5^ Environmental Sensing Systems Bentleigh East Victoria 3165 Australia; ^6^ Department of Geographical Sciences University of Maryland College Park Maryland USA; ^7^ Laboratory of Geo‐Information Science and Remote Sensing Wageningen University Wageningen 6708 PB the Netherlands; ^8^ Hawkesbury Institute for the Environment, Western Sydney University Locked Bag 1797 Penrith New South Wales 2751 Australia; ^9^ CSIRO 564 Vanderlin Drive Berrimah Northern Territory 0828 Australia; ^10^ Climate and Earth Observation Group National Physical Laboratory Hampton Road, Teddington London UK; ^11^ School of Natural and Environmental Sciences, Newcastle University Newcastle Upon Tyne NE1 7RU UK

**Keywords:** Automation, ecosystem structure, essential biodiversity variables, lidar, monitoring, vegetation structure monitoring

## Abstract

Climate change and increasing human activities are impacting ecosystems and their biodiversity. Quantitative measurements of essential biodiversity variables (EBV) and essential climate variables are used to monitor biodiversity and carbon dynamics and evaluate policy and management interventions. Ecosystem structure is at the core of EBVs and carbon stock estimation and can help to inform assessments of species and species diversity. Ecosystem structure is also used as an indirect indicator of habitat quality and expected species richness or species community composition. Spaceborne measurements can provide large‐scale insight into monitoring the structural dynamics of ecosystems, but they generally lack consistent, robust, timely and detailed information regarding their full three‐dimensional vegetation structure at local scales. Here we demonstrate the potential of high‐frequency ground‐based laser scanning to systematically monitor structural changes in vegetation. We present a proof‐of‐concept high‐temporal ecosystem structure time series of 5 years in a temperate forest using terrestrial laser scanning (TLS). We also present data from automated high‐temporal laser scanning that can allow upscaling of vegetation structure scanning, overcoming the limitations of a typically opportunistic TLS measurement approach. Automated monitoring will be a critical component to build a network of field monitoring sites that can provide the required calibration data for satellite missions to effectively monitor the structural dynamics of vegetation over large areas. Within this perspective, we reflect on how this network could be designed and discuss implementation pathways.

## Graphical abstract

Ecosystem structure is at the core of essential biodiversity variables. Spaceborne measurements can provide large‐scale insight into monitoring the structural dynamics of ecosystems, but they generally lack consistent, robust, timely and detailed information regarding full three‐dimensional vegetation structure at local scales. Within this perspective, we argue that establishing a global network of 4D monitoring using automated laser scanning of vegetation structure, StrucNet, should be a high priority within the research community.

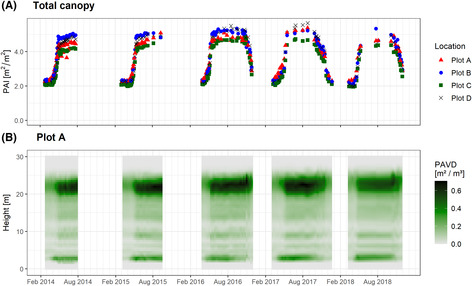

## Introduction

Biodiversity is under increasing pressure, with approximately 25% of animal and plant species currently threatened with extinction (IPBES, 2019). The main drivers of biodiversity loss include the removal of habitats, climate change and resource extraction pressures (Bowler et al., 2020; Pimm et al., 2014). The United Nations Sustainable Development Goals aim to address global biodiversity challenges through ambitious, community‐led conservation and restoration interventions, in particular for habitats and associated ecosystem service provisions. This acknowledges that natural habitats are necessary for the maintenance of biodiversity (Maron et al., 2018; Sinclair et al., 1995). Healthy ecosystems provide resources and services essential for a range of economic activities supporting livelihoods, food and water security (Díaz & Malhi, 2022). Loss of healthy ecosystems can impact human health directly (pests and diseases) or indirectly (water and soil quality) and can amplify the effects of climate change in both urban and rural areas (Muluneh, 2021; Shin et al., 2022).

The development of essential biodiversity variables (EBVs) provides a framework and set of indicators for monitoring biodiversity (Scholes et al., 2012; Skidmore et al., 2021). This framework has been developed to provide a reliable, technically feasible and economically viable tool for evaluating the effectiveness of interventions aimed at reversing or halting biodiversity decline. EBVs are measurable indicators designed to capture critical scales and dimensions of biodiversity with the ability to measure change through time. Within the context of this paper, we focus on vegetation ecosystems such as forests and savannas. Their ecosystem structure (e.g. ecosystem live cover fraction, ecosystem vertical profile) and function (e.g. primary productivity, vegetation phenology) are two of the six currently recognized classes of EBVs (https://geobon.org/ebvs/what‐are‐ebvs/). They can be measured using remote sensing and coupled with EBV classes relating to species and species traits (Valbuena et al., 2020), making them attractive to ecologists and conservation practitioners.

Ecosystem structure and function are often linked (Calders, Phinn, et al., 2020). Forest canopy structure affects movements and abundance of (semi)‐arboreal species, shaping resource and habitat availability, connectedness (Deere et al., 2020; Gámez & Harris, 2022; McLean et al., 2016) and microclimate (Boyle et al., 2021; De Frenne et al., 2013). Plant area volume density (PAVD, in m^2^/m^3^) vertical profiles in forests or savannas provide a direct measure of the degree of plant material occupying the vertical niche space (Marselis et al., 2019). PAVD profiles capture the complexity of vertical strata within the vegetation and are therefore directly related to EBV ecosystem vertical profile and biodiversity in general. PAVD has been linked to tree species richness across savannas to disturbed old‐growth forests in Gabon (Marselis et al., 2019), but also diversity of small mammals in forests of the Brazilian Cerrado (de Camargo et al., 2018) and Wisconsin, US (Schooler & Zald, 2019). Plant area index (PAI, in m^2^/m^2^) refers to half of the surface area of all aboveground canopy elements per unit of horizontal ground surface area and is the vertically integrated PAVD. Leaf area index, the foliage component of PAI, exerts major controls on exchanges of water, gas (including carbon dioxide) and energy in forest canopies.

Spaceborne remote sensing systems specifically designed for vegetation structural assessment (e.g. NASA Global Ecosystem Dynamics Investigation GEDI, ESA BIOMASS and NASA/ISRO NISAR) have the potential to provide insight into biodiversity at global scales (Skidmore et al., 2021). Their utility in assessments of change in ecosystem structure (e.g. biomass, extent, height, fragmentation) and threats (e.g. logging, infrastructure expansion) to biodiversity and ecosystems has been embraced by the science community, with high‐impact research demonstrating global patterns of change (McDowell et al., 2020). However, the interpretation of satellite data and derived metrics for EBVs, especially in the context of programmes for monitoring biodiversity at global scales, has been shown to be challenging. This is due to spatial scale effects (Comber & Wulder, 2019; Disney et al., 2019; Marselis et al., 2022), non‐frequent sampling (Valbuena et al., 2020) and sensor data limitations (Pennisi, 2021).

The EBV ecosystem vertical profile can be quantified as PAVD from a single observation; however, other EBVs such as ecosystem phenology or monitoring the impact of and recovery from disturbances (Bright et al., 2019) require a time series of observations (De Keersmaecker et al., 2022). Interpretations of satellite sensor data are also highly dependent on the availability of accurate and co‐incident ground reference measurements used for the calibration and validation of metrics generated from data acquired by the sensor (Duncanson et al., 2020). The sites used for collecting calibration measurements should be representative of ecosystems at a global scale with sufficient numbers and lifespans to allow the detection of gradual changes in space and time. For EBVs related to structure and function, such *in situ* data would ideally capture full three‐dimensional structure and dynamics at local scales to allow capturing the forest structural complexity relevant for biomass distribution, canopy density and connectedness (Ehbrecht et al., 2021), driving vegetation ecosystem functions and ecosystem services (Calders, Phinn, et al., 2020). *In situ* measurements of PAI and PAVD have the ability to capture small but significant shifts in structural dynamics (Calders, Origo, Disney, et al., 2018), thus allowing for quantification of temporal and spatial trends of EBVs. Previous work has demonstrated the ability of terrestrial laser scanning (TLS, also called terrestrial lidar) to capture forest structure and its dynamics (Smith et al., 2019; Stark et al., 2012). Recent advances in TLS sensors and algorithms have allowed us to monitor three‐dimensional vegetation structure with high spatial detail (Calders, Adams, et al., 2020). Typical measurements with TLS are mainly focused on acquiring data at a single point in time (e.g. EBV Ecosystem Vertical Profile) or non‐frequent revisits of sites. However, some studies have demonstrated the use of TLS for monitoring vegetation structural change with a high‐temporal frequency (Calders et al., 2015; Campos et al., 2020; Nunes et al., 2022).

Within this paper, we demonstrate the potential of high‐frequency terrestrial lidar remote sensing to monitor vegetation structural change at the timescales of processes driving that change. We illustrate how a 5‐year time series of TLS data can be used to derive important metrics of forest structure related to key EBVs, and for calibration and validation of satellite‐based global vegetation structure monitoring. We then discuss the limitations of traditional TLS systems for temporal studies and present an automated structural monitoring concept based on a network of field monitoring sites on a global scale. We identify the goal of a global network here because the protection of our vegetation ecosystems and the biodiversity they support require a global view and hence a systematic global network of calibration sites.

## Proof of concept: high‐temporal TLS

Approaches for analysing three‐dimensional TLS data from vegetation can be broadly categorized into (1) explicit geometrical modelling and (2) turbid medium methods (Newnham et al., 2015). The latter is typically used to estimate PAI and PAVD using pulse‐based (Calders et al., 2014; Jupp et al., 2009) or voxel‐based (Béland et al., 2014; Pimont et al., 2018) approaches, and are well‐suited for objective and automated processing of large amounts of structural data related to EBVs. Both turbid medium approaches have been extensively used to characterize vegetation canopies and are implemented in open‐source libraries such as pylidar (www.pylidar.org) or AMAPVox (Vincent et al., 2017).

Vegetation phenology typically encompass a permanent signal (‘background signal’) and a variable signal that corresponds to seasonal dynamics (Clerici et al., 2012). This variable signal can be characterized by an initial growing period (e.g. leaf emergence), a maturity period (maximum leaf area) and a senescence period (e.g. leaf senescence or abscission). As such, phenology determines, for example, carbon uptake by trees, crop yield potential, plant competition and resource availability for wildlife and surface albedo and microclimate. We recently demonstrated the potential of TLS data, acquired with a RIEGL VZ‐400 survey‐grade TLS instrument at four sampling locations in Dassenbos, a broadleaf deciduous forest in Europe (Wageningen; the Netherlands; 51.9829°N, 5.6558°E) to monitor spring phenology and quantify structural impacts from storm damage (Calders et al., 2015). TLS measurements of structural change generally corresponded well with field observations of leaf phenology, overcoming limitations of MODIS‐derived NDVI time series, which showed a lag to detect the start of the season (7–12 days). TLS measurements were also capable of separating structural changes of tree canopies and understory (Calders et al., 2015; Nunes et al., 2022), which is not possible to do directly using satellite measurements from passive sensors.

**Figure 1:**
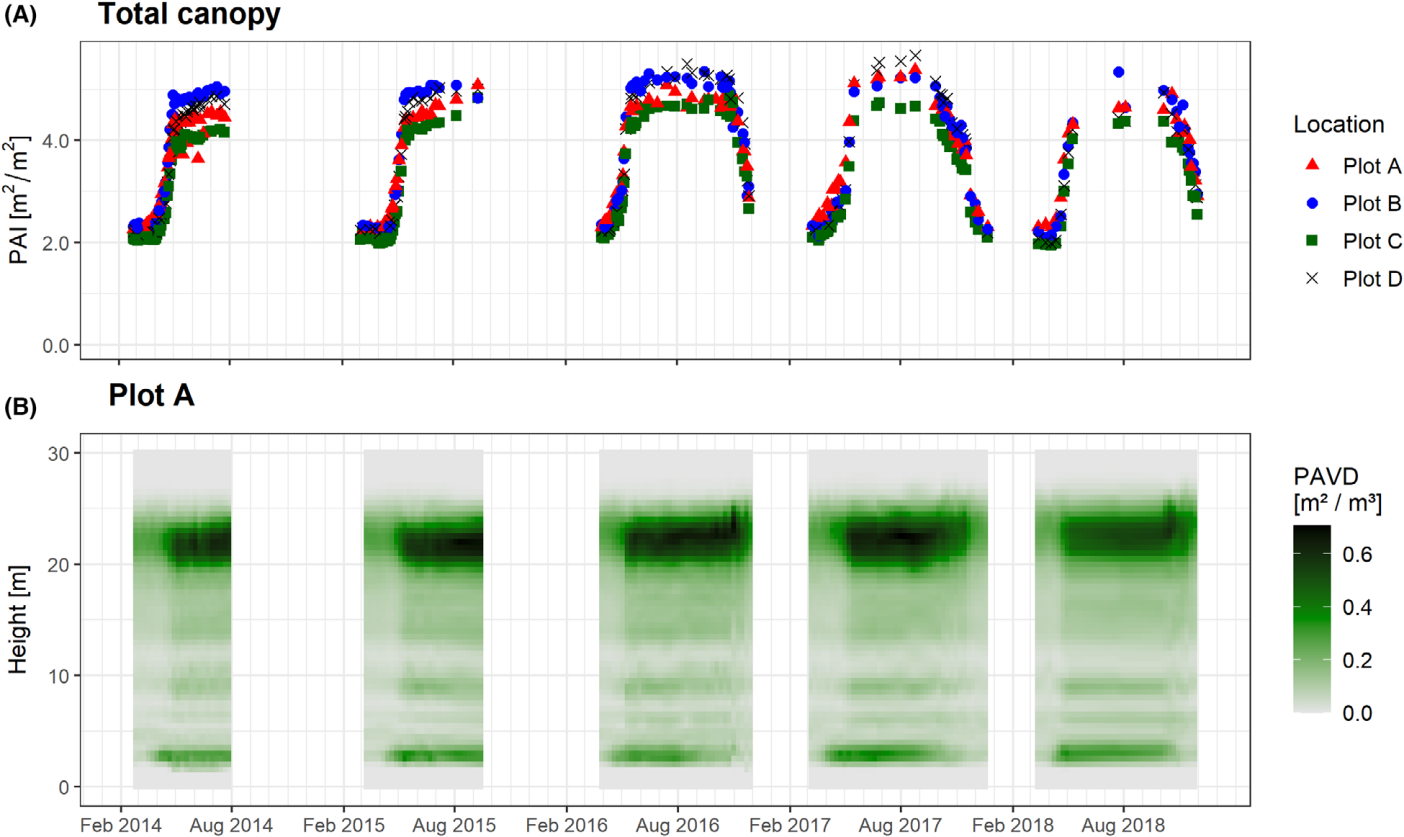
Five‐year dynamics of forest structure for the four sampling locations in Dassenbos. Data were collected using the same measurement protocol and data analysis as described in Calders et al. (2015) using a zenith range of 35–70° for 184–186 (some scans were discarded for quality purposes) measurement days during the period from February 2014 to November 2018. Panel (A) represents total canopy plant area index (PAI) estimates derived from a terrestrial laser scanning (TLS) vertical plant profile. Panel (B) shows plant area volume density (PAVD) for each measurement at sampling location A.

Continued repeat measurements over a 5‐year period at the same four sampling locations consolidated the potential of TLS for monitoring phenology (Fig. 1). Furthermore, the extended data collection periods in 2016, 2017 and 2018 demonstrated new opportunities, such as capturing the start and end of the growing season, from which one can calculate its duration. The latter could prove invaluable for monitoring climate change effects on vegetation senescence at regional scales, for which evidence remains inconclusive or sparse (Gill et al., 2015). It could therefore provide complementary information to existing measurements such as the PhenoCam network (Brown et al., 2016; Wingate et al., 2015). However, our time series also illustrates the practical limitations of this approach using TLS. This is mostly due to the practical limitations of surveying TLS instruments such as power requirements, the need for a field crew or the robustness of re‐measuring the same location. The relatively high purchase costs can be difficult to justify for dedicated high‐temporal measurements at a single site (or a few sites in close proximity) only. Restrictions in instrument availability are also present in our data, where periods with no data correspond to periods when instruments were unavailable. Furthermore, TLS instruments are often over‐designed for the purpose of gap probability analysis and sensors with more sparse angular sampling can be used (Calders et al., 2014) as long as the onboard laser is powerful enough to exit the canopy. Whereas our 5‐year TLS time series illustrates the potential of active ground‐based monitoring, the practical limitations discussed mean that this approach is currently not scalable for establishing a global network for high‐temporal monitoring of structural dynamics of forests.

## Automated high‐temporal laser scanning is necessary to build a global‐scale network for vegetation structure monitoring

To make ground‐based monitoring feasible at a network scale, automated and cost‐effective high‐temporal laser scanners are needed to overcome the practical limitations of repeat monitoring of vegetation with TLS sensors. Eitel et al. (2013) demonstrated a prototype for automated laser scanning. The first automated laser scanning instrument designed for permanent outdoor deployment was the VEGNET *In‐Situ* Monitoring Lidar (IML) (Culvenor et al., 2014; Griebel et al., 2015; Portillo‐Quintero et al., 2014). This IML sensor used an off‐the‐shelf phase‐based laser rangefinder and scanned at a fixed zenith angle, Θ, of 57.5°. At this ‘hinge angle’ of 57.5°, the foliage angular projection function G(Θ) is essentially invariant at 0.5 over different leaf angle distributions (Ross, 1981), allowing for straightforward calculation of PAI using directional gap probability‐based methods such as those described by Jupp et al. (2009). A major limitation of the VEGNET IML sensor was the maximum range of 60 m, which precluded monitoring very tall forests, and the low reflectance of foliage at the laser wavelength of 635 nm. The range is important because laser pulses need to ideally be able to escape the canopy to correctly calculate the gap fraction and derived vertical plant profiles.

**Figure 2:**
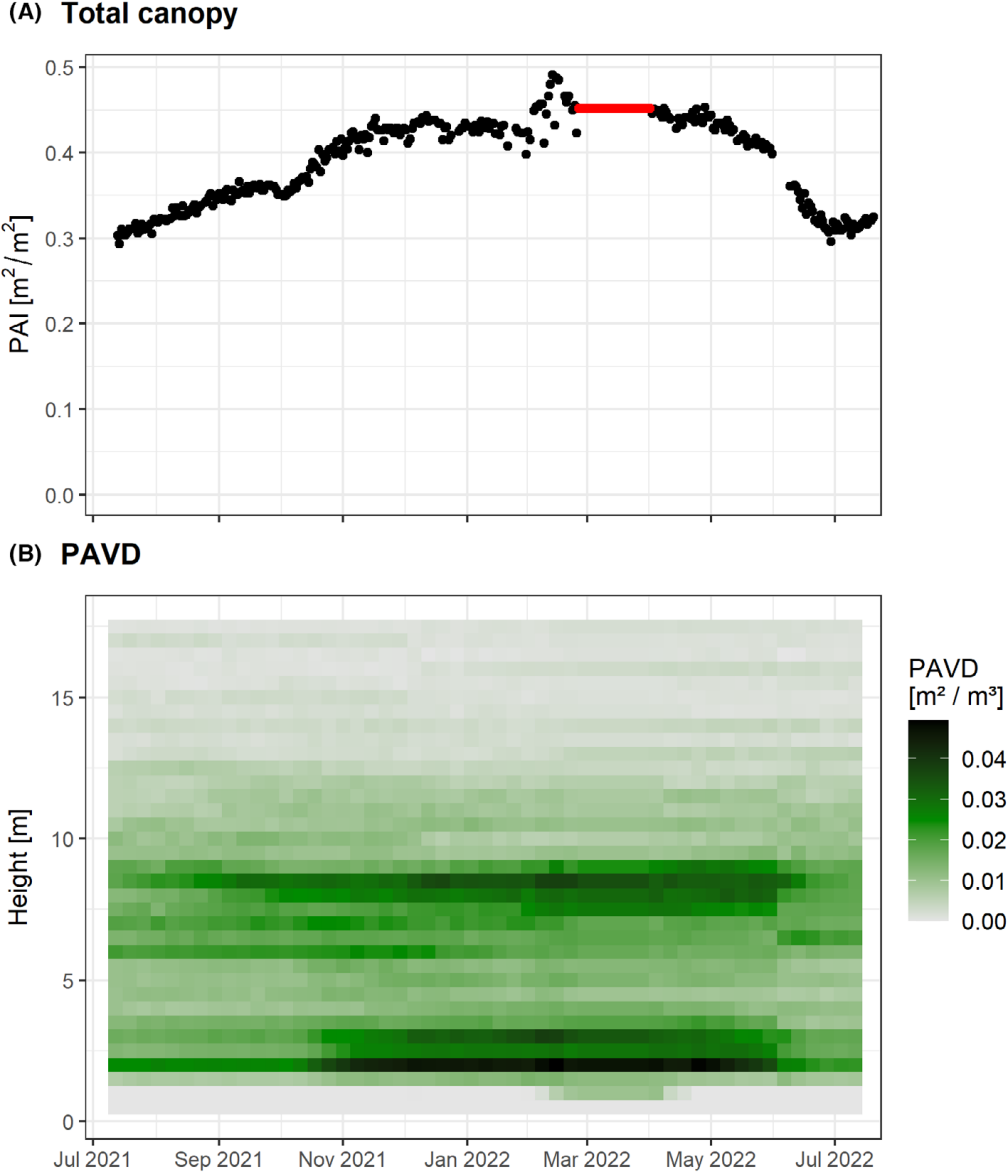
Time series of (A) total canopy plant area index (PAI) and (B) plant area volume density (PAVD) from high‐temporal laser scanning at a tropical savanna site in Northern Australia. Planned fuel reduction burns were conducted in May 2021 and June 2022. LEAF sensor was installed in mid‐July 2021. Data showing from installation until 20 July 2022. Of the 368 hemispherical scans acquired, 64 scans were identified as rain affected and removed from the analysis. PAVD profiles were calculated for the remaining 304 hemispherical scans at 0.5 m height increments. Gaps in the time series were filled using linear interpolation and then averaged at a weekly timescale for visualization, that is each ‘square’ in the PAVD plot (B) represents 0.5 m in height and 1 week in time. All scans during March were rain affected and discarded with PAVD (and derived PAI) assumed to be static during this time (red line).

Recent developments in sensors overcome the range limitation of the discontinued initial VEGNET IML. For example, the LEAF sensor has an extended range of 100 m, as well as improved protection against dust and rainfall. It uses a 905 nm time‐of‐flight laser with beam divergence of 5 mrad and has a hemispherical scan range of 0–360° in azimuth and 0–130° in zenith. Ancillary sensors for monitoring instrument status increase the success of long‐term deployments, such as an internal humidity sensor to alert water ingress, and a tilt sensor for detecting movement of the sensor tripod, for example, from wild animals or unstable ground following rain. In Figure 2, we demonstrate the use of a LEAF sensor at a tropical savanna site near Darwin, in Northern Australia. The region has a tropical climate with a distinct wet season (November to April) and dry season (May to October). The field site is characterized by a sparse Eucalypt overstorey with sub‐strata of small trees, shrubs and tall grass. The site had a planned fuel reduction burn in May 2021, resulting in the removal of grass fuel and some loss of foliage from fire‐tolerant trees. One LEAF sensor was installed in mid‐July 2021 and was configured to acquire daily hemispherical scans with an angular resolution of 1.8° in azimuth and zenith, resulting in 14 400 laser samples (‘shots’) and scan duration of 14 min. To maximize signal‐to‐noise ratio, scans commenced at 9 pm local time, that is at a time of negligible solar illumination. The instrument was removed on 31 May 2022 while another fuel reduction burn was conducted. It was reinstalled 1 week later at the same location and another 6 weeks of data acquired for inclusion in analysis. The dataset presented therefore spans 12 months and captures the structural impact and response to two fuel reduction burns.

From July to November 2021, Figure 2b shows a relatively static understorey and overstorey and a gradual increase in PAI in the mid‐stratum from 6 to 9 m. Commencement of the wet season in November was associated with a sudden increase in PAI from understorey vegetation (1.5–4 m) and an accelerated increase in PAI through the mid‐stratum. The low‐stratum and mid‐stratum trees are more shallow‐rooted than the overstorey trees and therefore more dependent on seasonal rains for growth. These trees and shrubs will also have been more affected by the fuel reduction burn than the overstorey trees and thus may exhibit a more notable increase in aboveground biomass under favourable growing conditions.

**Figure 3:**
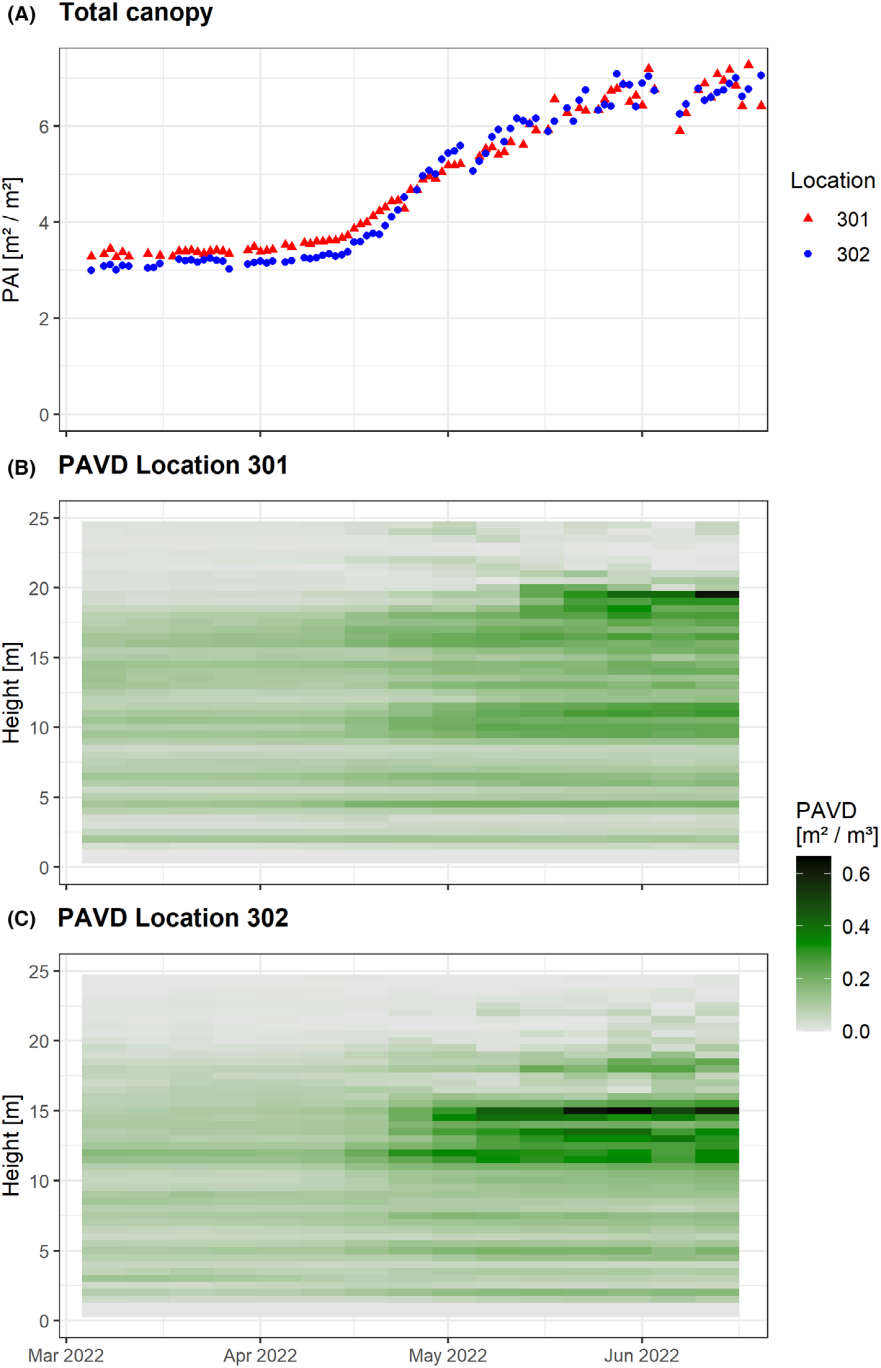
Time series of (A) total canopy plant area index (PAI) and (B, C) plant area volume density (PAVD) from high‐temporal laser scanning with LEAF at two locations in Wytham Woods, UK, from 5 March until 22 June 2022. Of the 110 scans acquired by each instrument, 20 scans were rain affected and discarded from further analysis. PAVD profiles were calculated for the remaining 90 hinge angle scans at 0.5 m height increments. Gaps in the time series were filled using linear interpolation and then averaged at a weekly timescale.

Figure 3 demonstrates the co‐deployment of two LEAF sensors in Wytham Woods, UK (https://www.forestgeo.si.edu/sites/europe/wytham‐woods). The forest is a typical temperate forest site in southern Great Britain (Kirby et al., 2014) and is dominated by three deciduous species, *Fraxinus excelsior*, *Acer pseudoplatanus* and *Corylus avellana*. The sensors were installed in early March 2022 so they could simultaneously capture the spring phenology in close proximity, which typically results in an ‘S‐curve’ that is characterized by a period of rapid growth and convergence towards a stable plateau of maximum PAI (Calders et al., 2015). The aim here was similar to phenology monitoring in Dassenbos (Fig. 1), with an interest in acquisitions from multiple instruments located in separate plots with near proximity, thereby eliminating between‐plot variability due to sequential acquisitions and potentially different background illumination and weather conditions. The LEAF instruments were configured to acquire daily ‘hinge’ angle scans, consisting of five azimuth scans, or ‘rings’, centred on 57.5° zenith with an angular separation in zenith of 0.45°. Angular resolution in azimuth was 0.067°, resulting in 27 000 shots over a period of 21 min. Scans commenced at 10 pm local time irrespective of weather conditions. The data show interesting differences in PAI time series at the two plot location within Wytham Woods. Sensor 302 has an overall lower PAI in leaf‐off conditions that continued until late April. Sensor 302 sees a larger increase in PAI compared to sensor 301, and Figure 3b and c suggests that this is mainly driven by a higher leaf onset in the canopy layer between 10 and 15 m.

Irrespective of which automated lidar sensor is used, filtering data due to adverse weather will be a necessary step in time‐series analysis. At a forest site in southern Australia with a typical Mediterranean climate, Culvenor et al. (2014) discarded approximately 50% of their IML data due to wind and rain over a 1‐year period. In contrast, Griebel et al. (2015) discarded just 16% of data from the same instrument type due to high wind speeds and condensation over a 2‐year period, highlighting that local site conditions can exert strong influences on data quality and quantity. Dominant seasonal factors such as monsoonal rainfall also need to be considered. For example, while only 17% of scans were rain affected at our study site in Northern Australia (Fig. 2), the absence of any usable data during the high rainfall month of March resulted in a notable gap in the time series. Data loss from rainfall can partly be addressed by oversampling temporally (i.e. more than one daily acquisition) at the cost of increased demand for power and data storage. Another solution currently being evaluated involves the use of rainfall sensors to reschedule scans if rainfall is detected prior to a scan commencing.

## Outlook and conclusion

Past work using passive sensors demonstrated the value of global datasets of forest structure (Pfeifer et al., 2018) or the use of automated measurements using digital hemispherical photography (Brown et al., 2020). Within this paper, we demonstrated the added value of the third spatial dimension (detailed vertical profiling) to structural monitoring through active ground‐based sensors, and the addition of the fourth dimension, time (monitoring vertical structural changes), through automated monitoring. These measurements enable the study of vertical structural dynamics of vegetation by separating multiple vegetation layers, which have been demonstrated to play an important role in defining 3D niches in the context of habitats and biodiversity (Gámez & Harris, 2022). A study of forest leaf area in the Amazon by Smith et al. (2019) showed how compensating shifts in overstorey and understorey dynamics resulted in a net neutral PAI estimate for all strata combined, emphasizing the importance of vertical structural information for describing plant phenological strategies in mixed species environments. Similarly, the ability to monitor canopy layers separately (as opposed to full canopy‐integrated metrics) supports inferences about where and how seasonal dynamics are taking place in the canopy (Griebel et al., 2017; Nunes et al., 2022). These continuous observations can also be related to measurements from active airborne and spaceborne sensors including LiDAR waveforms and tomographic SAR backscatter profiles (Fatoyinbo et al., 2021).

**Figure 4:**
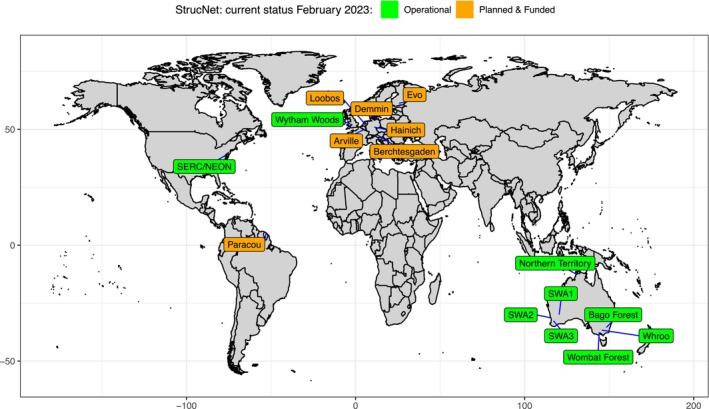
Overview of StrucNet sites, status February 2023. This includes nine operational sites (some sites also have multiple instruments), seven sites that have LEAF sensors planned and funded. Currently, two sites have funding for sensors pending (Aelmoeseneiebos, Belgium and Yangambi, Democratic Republic of the Congo).

Establishing a global network of 4D monitoring of vegetation structure, StrucNet, should be a high priority within the research community. Conserving biodiversity in vegetation requires a global view and hence a systematic global network of calibration sites. This is similar to the drivers that led to the establishment of FLUXNET, a global network of micrometeorological tower sites (Baldocchi et al., 2001). StrucNet would pair with existing global vegetation monitoring networks (e.g. PhenoCam, ForestGEO) and fill a critical data gap by adding vertical vegetation structure, but also be adaptive to the local requirements of land management agencies (e.g. post‐fire recovery monitoring). It will provide an essential link between more traditional vegetation measurement techniques and the increasingly detailed and accurate remotely sensed signals from *in situ* (e.g. FluxNet), airborne (e.g. NEON Airborne Observation Platform) and spaceborne (e.g. NASA GEDI) instrumentation. StrucNet will not only support calibration and validation of remote sensing data, it will also provide data on the impact of climate change on plant phenology (Piao et al., 2019) and forest disturbances and recovery (e.g. fire impacts; see Fig. 2) through objective 3D time series at scales relevant for planning and decision‐making on management and interventions. The examples shown in Figures 2 and 3 demonstrate how these 4D measurements can be important to monitor EBVs related to the structure and functioning of vegetation ecosystems. Whereas we have demonstrated that LEAF sensors can be used to build this global network, this could essentially be any instrument that fits the operation and data criteria, that is automated, unattended operation and multi‐angular vertically resolved structural measurements at a plot scale. Currently, a number of research sites, in practice early StrucNet adopters, have been equipped with LEAF sensors already or will be equipped in the near future as part of newly funded projects (Fig. 4). Despite these early adopters, several areas of the world still lack adequate coverage, and the establishment of additional StrucNet sites should aim to overcome these data gaps.

The advancements in hardware and algorithms for automated processing of 3D data in the past two decades have now made it possible to deploy automated lidar sensors for monitoring EBVs across a wider vegetation plot network. The success of global networks like FLUXNET, the PhenoCam network and national meteorological networks is defined by standardized protocols for instrument setup, data collection and processing, instrument intercomparison and, if possible, calibration to a common standard. In the case of essential climate variables, these standards are often based on traceability to the international system of units (SI), which guarantees a universal and constant measurement framework over time. As vegetation vertical structure was recently declared an EBV (Skidmore et al., 2021), there is an urgent need to establish such standards to reliably quantify the change in EBVs within and across terrestrial ecosystems. In particular, traceability to a common standard or intercomparison has priority as there is already a range of lidar instruments with different laser and sampling properties at different scales available such as terrestrial, mobile, drone and airborne laser scanning, plus a range of retrieval methods. All of them allow the estimation of vegetation structure but with unquantified uncertainties and biases. In this context, calibration with direct, destructive measurements is often labour‐intense (Béland et al., 2011) and impossible at permanent monitoring plots. However, relatively efficient procedures have been proposed to link direct measurements of small canopy volumes to TLS, and take into account scanner properties and setup (Pimont et al., 2015; Soma et al., 2018). With the anticipated future development of a range of automated high‐temporal laser scanning sensors, benchmarking exercises as well as the use of radiative transfer modelling (Calders, Origo, Burt, et al., 2018) will be key in the required interoperability of automated 4D data collected across a global plot network.

## Author Contributions

KC, BB, GN and MH, JA and DC conceived the ideas and designed methodology; KC, BB, GN, DC, JA, HB, JH, AL, SL, RM, NO and JV collected the data; KC, BB and DC analysed the data; KC led the writing of the manuscript. All authors contributed critically to the drafts and gave final approval for publication.

## Conflict of Interest

The authors declare no known conflicts of interest. DC is employed by Environmental Sensing Systems and developed the LEAF sensors. The manuscript discloses that essentially any instrument that fits the operation and data criteria can be used in StrucNet.

## Data Availability

The TLS data and LEAF vertical profile data are available at 10.5281/zenodo.7778743.
